# On the Induction of Premature Labor — Modes of Effecting It

**Published:** 1855-07

**Authors:** 


### ECLECTIC DEPARTMENT,

### AND SPIRIT OF THE MEDICAL PERIODICAL PRESS.

---

*On the Induction of Premature Labor — Modes of Effecting it.*—The following communication, received from Dr. H. R. Storer, will form a part of Prof. Simpson's forthcoming work, and is now for the first time published:

A variety of means or plans have been proposed for the artificial induction of premature labor, in those various and important complications which are now so generally recognized by the obstetric profession as demanding this mode of operative interference.

Thus it has been attempted to excite the uterus into parturient action:

1. By external abdominal frictions, so as to irritate its outer surface.
2. By passing currents of electricity or galvanism through its walls.
3. By irritating other, and even distant, parts or surfaces, as the vagina, rectum, or nipple, that are known to possess a marked reflex power over the contractility of the uterus.
4. By the internal exhibition of ergot of rye and other oxytoxic remedies.
5. By the evacuation of the liquor amnii.
6. By the dilatation of the os uteri.
7. By the separation of the membranes from the cavity of the cervix or body of the uterus by the finger, by instruments or sponges, or by the injection of fluids.

The three first of these modes of inducing premature labor are—alone and singly—so very uncertain in their results, and so generally and entirely fail, that few or no accoucheurs place any confidence in them;* and to the fourth the same objection applies, with this addition, that the ergot, even when it has succeeded, has proved too dangerous in its effects upon the child to be used in an operative procedure, instituted, as this usually is, for the very purpose of saving the infant.

The fifth mode which we have enumerated above, viz., the evacuation of the liquor amnii, is, of all the methods proposed, both the oldest and assuredly the most sure and fixed in its effects. But, as a common means, and

---

* Several years ago I attended a case with Dr. Thatcher, in which he applied a child to the breast with the object of exciting pains. Some hours before, I had introduced a large sponge-tent into the os uteri. There was a wet-nurse in attendance to suckle our patient's infant as soon as it was born. It was the nurse's child which we applied to the nipples; and, as she thought, with the effect of increasing the uterine contractions and pains, which had already begun to appear. I have never, however, seen such an application of an infant to the nipples *originate* uterine contractions, nor in the two or three cases in which I have tried the plan of Schoeller and Braun, of distending and consequently irritating the walls of the vagina with masses of sponge or a dilating caoutchouc bottle, have I been at all successful in exciting the uterus to parturient action. I have not seen the abdominal frictions of D'Outrepont and Ulsamer tried.—*On Galvanism, see page* 376.

when labor is induced to save the infant, it is liable to one strong objection, viz., that it is undoubtedly much more dangerous to the child than the employment of operative procedures, which—as the dilatation of the os, or the separation of the membranes—allow the bag of membranes to remain entire, and thus keep the fragile and premature infant protected by the amniotic fluid during the progress of the labor, or at least during the earlier stages of it.

In by far the greater number of instances in which I have had occasion to induce premature labor in private and consultation practice, I have always, in the first instance, avoided the artificial evacuation of the liquor amnii, and have proceeded upon the principle either

I. Of dilating the cervix uteri; or,

II. Of separating the membranes.

Or rather I have acted upon both of these plans conjointly, for it is difficult or impossible to follow out thoroughly the one indication without, in some respect at least, following out the other also.

### I. DILATATION OF THE OS AND CERVIX UTERI.

In exciting premature labor upon this principle, accoucheurs have used three different means:

1. The finger.
2. Metallic dilating forceps and instruments. And
3. Sponge-tents.

To stretch, however, and open the os uteri by the finger or by metallic dilators, is a process so irritating and painful, that few or no practitioners now use it; especially as the same object can be effected more easily and safely by the introduction of compressed sponge.

*Sponge-tents* were first proposed as a means of inducing premature labor, by Kluge and Brunninghausen: and they have been much employed for the purpose both in Germany and France. All the continental accounts, however, of their employment, up even to the present day, describe the introduction of the tents into the os uteri as a complicated operation, requiring always the aid of the speculum, and the use of a vaginal tampon, or other means, to keep the tent in situ. But there is no necessity, whatever, for such formidable arrangements.

In 1844, when first mentioning the induction of premature labor in this country by sponge-tents, I attempted to show that they could be easily introduced and employed without any vaginal speculum or tampon, or in the simple mode already described in a preceding paper on Intra-Uterine Polypi (see p. 127). And for several years subsequent to that date, I had recourse to this mode of inducing premature labor in a long series of cases; always with perfect success as regarded the mother, and in a large proportion of cases with safety also as regarded the child.

I never found this means fail, although in a few instances I have seen the dilatation effected to the size of a half-crown or more, for thirty or forty hours before true uterine contractions set in. Generally, however, parturient action began long before the dilatation of the os uteri had reached these dimensions; and when it did so, a considerable part of the first stage of labor was thus, as it were, found finished before actual labor commenced. Sometimes uterine pains and contractions began as early as four or six hours after the sponge-tent was introduced, especially if the tent were of considerable size, and means were used for its rapid development. In almost every case, the first tent employed may be as thick as the little finger; and the patient should be directed to have injected into the vagina every hour or two, a small quantity of warm water for the imbibition and expansion of the compressed sponge. She should lie on the back during, and for some time after, each injection, in order that the water may be more thoroughly retained. After the first sponge is fully dilated, it may be withdrawn, and a second and larger one introduced; or, without removing the first, tents of a greater and greater size may be introduced at intervals of six or eight hours, till the os uteri is thoroughly dilated or labor supervenes.

To the induction of premature labor by the use of sponge-tents, I have heard some accoucheurs object, on the ground that, from want of practice, they have had difficulty in introducing the compressed sponge into the os uteri. A much more important drawback to the method will be found in the circumstance, that the presence of a large sponge-tent in the canals of the cervix uteri and vagina, sometimes, as a foreign body, produces such a degree of local uneasiness and irritation, as to inflict no small amount of discomfort and continuous pain upon the patient. It is principally on this account, and to avoid this difficulty, that, of late years, I have in my own practice commonly brought on premature labor by the other means already alluded to, namely, the detachment of the membranes—a process not requiring the permanent retention of any material in the maternal passages, and capable of being effected with probably less difficulty and trouble to both practitioner and patient.

### II. SEPARATION OF THE MEMBRANE.

In the induction of premature labor, the membranes of the ovum have been proposed to be mechanically separated from the interior of the uterus, by different means, to different degrees, and in different localities.

The idea that the partial artificial separation of the membranes would lead on to labor occurred first to the late Professor Hamilton; and he was himself the first also to put it in practice as far back as 1795.

*Dr. Hamilton's Method, by the Finger, &c.*—In operating, he detached "a portion of the decidua from the cervix uteri," by the introduction, first, of his finger, and ultimately of a bent brass wire. His friend Dr. Burns describes Dr. Hamilton's operation as consisting of "insinuating a finger within the os uteri, and gently dilating it, and detaching a part of the membranes from the portion of the cervix in its immediate vicinity." "If," he continues, "we have not thought it prudent to dilate at once the os uteri, so as to admit the finger freely to touch the membranes, we may repeat the dilatation gently at the end of a few hours, and then detach the membrane cautiously from the cervix uteri by the finger to the extent, perhaps, of two inches. But for this purpose," Dr. Burns adds, "it may be necessary, if the os uteri be high, to have the *hand* introduced into the vagina; or sometimes the detachment has been accomplished with a catheter or other small instrument." As thus pursued, this mode of inducing labor by separating the membranes from the cervix, was not always unaccompanied with pain, particularly when the fingers, and especially the hand, were introduced; it was very tedious, and sometimes it failed, as Dr. Hamilton himself states, and the operation required to be completed by puncture of the membranes and evacuation of the liquor amnii.

*Dr. Kiwisch's Method, by Injection of Water.*—In 1846, Professor Kiwisch proposed to bring on premature labor, by injecting a stream of tepid water into the vagina, and against the cervix and os uteri. His apparatus, as delineated by Scanzoni, consists of a small square tin box or reservoir of water, fastened to the wall at the height of nine or ten feet, and from the bottom of this reservoir a tube hangs down, the end of which is, when required, introduced into the vagina, so as to allow a strong continuous stream to pour through it, against the cervical portion of the uterus.

The douching or injection was recommended to be repeated morning and night, and commonly labor supervened on the fourth or fifth day.

This plan of Dr. Kiwisch's was shortly afterwards tried successfully in Vienna, Berlin, &c., by various Continental practitioners. In April, 1851, I described a case to the Edinburg Obstretic Society, in which I used this method. It was an instance where the patient had repeatedly found the child die a short time after quickening, and retained it for six or eight weeks subsequently. During her last pregnancy, the same occurrence took place with the same symptoms. A few weeks having elapsed, she threw up tepid water at my request, twice a-day, with the view of bringing off the dead fœtus. After nine douches, applied night and morning with a common syringe, expulsive pains came on, and a dead and shrivelled fœtus and placenta were expelled. In the course of that and the subsequent years, I had various opportunities of bringing on premature labor by the same means, and, as I always found, with almost perfect certainty as to the power of its induction.

Professor Kiwisch imagined that the vaginal water injection induced labor by the imbibition of the fluid relaxing the soft parts. The flow of a gentle and small stream of water into the vagina ought, if this were the true principle, to act as well as a stronger current. But a short experience convinced me that this was not the fact; and it soon became evident—1. That the water douche was liable to fail, unless the injected fluid accumulated and distended the vagina, so as to expand that canal and enter the os uteri; and 2. It seemed the more rapid and certain in its action, in proportion as it entered freely into the uterine cavity itself, and in proportion, therefore, as it separated more of the surface of the fœtal membranes from the interior of that cavity.

In only two or three cases did I try an elevated box and syphon tube, like that originally suggested by Kiwisch. From the first, I found a common enema syringe a far better and more manageable apparatus. Usually I have employed the India-rubber syringe of Dr. Kennedy, or that of Mr. Higginson. At first I merely injected and distended the vagina, retaining the fluid in it by closing the vulva with pressure of the fingers or hand, and thus forcing the water upward through the os into the uterine cavity; but I soon found it a simpler and more direct plan to introduce the end of the syringe through the uterine orifice, and thus send the stream directly into the interior of the uterus, without unnecessarily distending the vaginal canal. In most cases it is easy to pass for this purpose the common ivory nozzle of the enema syringe through the os uteri; but when that opening is placed very high, or far backward, I have found that the addition of a longish gum-elastic pipe or bent silver catheter to the nozzle of the tube greatly facilitates the requisite introduction of the instrument through the os and upward for an inch or two, between the membranes and the anterior or posterior wall of the uterus.

While the practitioner is using the syringe and injecting the fluid, the patient should lie on her left side, and with the pelvis placed near the edge of the bed or sofa which she is occupying. A basin properly placed immediately below, both contains the water to be used, and receives it again after it re-escapes from the vulva. The tubes of the catheter and syringe should be carefully filled with the water before commencing the injection, lest a quantity of air be thrown into the uterine cavity. Usually the injection is carried to the extent of the patient complaining of a feeling of distension or fulness; and it may be repeated twice a-day, or oftener, according as it is an object or not to expedite as much as possible the supervention of labor.

It was not till I had used this method for a considerable time, and in a number of cases, that I discovered that a similar method had been suggested and described by Dr. Cohen of Hamburg.

In several cases where the child was placed with the head over the os uteri, I have found it change its position as the water injection proceeded, and an upper or lower extremity to present. Occasionally this preternatual presentation has remained; but more frequently the child has again rotated, and the head again become replaced over the uterine orifice. In no case have I seen any great amount of hemorrhage from partial separation of the placenta. But the repetition of the injection sometimes becomes irksome to the mother as well as to the accoucheur.

*Detachment of the Membranes, by the Uterine Sound, from a Portion of the Body of the Uterus.*—Believing that labor was, at the ninth month, induced naturally through the degeneration and loosening of the decidua (see p. 351), I was encouraged last year to try to induce it artificially by the mechanical separation of a portion of the membranes from the interior of the body of the uterus.

In general the stethoscope sufficiently certifies to us the locality of the placenta, and what part or side of the uterus we ought consequently to avoid;* and nothing in the way of an operation could possibly be more simple or more easy and painless than the introduction of a sound, through the dilatable os, and upward for five or six inches, between the membranes and the anterior wall of the pregnant uterus.

In the first case in which I tried this plan, the patient, after having been always delivered in the country by craniotomy, has thrice had premature labor induced under my care. Her three children are alive. On the first occasion, in 1851, she had an apparatus upon the plan of Kiwisch's erected; but it required to be used, and that frequently, for five or six days before labor supervened. On the second occasion, I injected a quantity of tepid water by an enema syringe into the uterine cavity, and the child was born

---

* In injecting water we have no control on the *direction* it will take in the uterine cavity, while we can regulate perfectly that of the sound. In one case, from inattention to the uterine souffle, I probably separated the edge of the placenta, as a clot was found at that spot. The child was born alive; and the mother recovered perfectly. But with due caution such an accident should be easily avoided.

in about twenty-four hours afterwards. Last year, on the third occasion, I saw her late at night along with my friend Dr. Ziegler, and passed a uterine bougie for five or six inches upward between the membranes and the anterior wall of the uterus. The child was born before noon next day. At the time of passing the bougie, the patient herself was not aware that anything special had been done, but believed that I was merely making a common digital examination, in order to ascertain the exact stage of pregnancy, &c.; and she subsequently declared, that, in her experience, this last method was too simple to be capable of being compared with the two other methods to which she had been formerly subjected. But in all cases, a single introduction of the bougie will by no means suffice. Like the tents and douching, it requires in most instances to be repeated more than once. During the past three month of the present year, I have induced labor six or seven times by this method. In one case, in my own private practice, and in another under the care of Dr. Scott, of Musselburgh, the labor was terminated within eighteen hours. In the others, parturition did not come on till the second or third day after the act of separation. In a case which I saw with Dr. Thomson, he used a water injection next day, and on the subsequent day I again separated the membranes with the bougie. Parturient action began that night. In a previous labor of this woman, the child was rotated, and made to present prematurely by the employment of the water injection. All the children have been born alive in the ten or twelve cases in which I have induced premature labor by the uterine sound.

The relative degree of facility or difficulty with which labor is induced artificially in different women, or even in the same woman in different pregnancies, varies very greatly. Where one plan fails, the addition of a second, or of a third method, will sometimes enable us to succeed; and if all modes less safe for the child prove ineffectual, as the separation of the membranes with an uterine bougie, the water injection, and a sponge-tent, we may always at last determine the certain occurrence of uterine contraction by the uterine puncture of the membranes. And if we have recourse to this puncture, we may still in a great measure save the liquor amnii for the protection of the child during labor by making the seat of the opening oblique and as high as four or five inches above the os, as recommended by Hamilton and Meissner. One of the best instruments for effecting this object is that long ago recommended by Dr. Hamilton, viz., a male catheter having an open or truncated extremity, and provided with a silver wire to pass through it for the puncture of the membranes. The membranes, I believe, will sometimes be found to rupture high up when and where they are simply separated from the body of the uterus by the introduction of the knobbed uterine sound or bougie.—(April, 1855.)—*From Boston Med. and Surg. Journal.*

